# Vaccination in Multiple Myeloma: Challenges and Strategies

**DOI:** 10.1111/ejh.70013

**Published:** 2025-07-26

**Authors:** Enrica Antonia Martino, Ernesto Vigna, Antonella Bruzzese, Nicola Amodio, Eugenio Lucia, Virginia Olivito, Caterina Labanca, Santino Caserta, Francesco Mendicino, Fortunato Morabito, Massimo Gentile

**Affiliations:** ^1^ Hematology Unit, Department of Onco‐Hematology Cosenza Italy; ^2^ Department of Experimental and Clinical Medicine University of Catanzaro Catanzaro Italy; ^3^ AIL Sezione di Cosenza Cosenza Italy; ^4^ Department of Pharmacy, Health and Nutritional Science University of Calabria Rende Italy

**Keywords:** immunosuppression, MM, vaccination

## Abstract

**Background:**

Multiple myeloma (MM) is a hematological malignancy characterized by profound immunosuppression resulting from both disease‐related mechanisms and treatment‐induced immune dysfunction. This compromised immune status markedly increases susceptibility to infections, a leading cause of morbidity and mortality in MM patients. While vaccination represents a cornerstone of infection prevention, standard immunization strategies often yield suboptimal responses in this population.

**Objectives:**

This review synthesizes current evidence on the immunological barriers and clinical effectiveness of vaccination in MM. We evaluate vaccines targeting influenza, *Streptococcus pneumoniae* , SARS‐CoV‐2, and other relevant pathogens, and explore determinants influencing vaccine efficacy, including optimal timing, formulation, and patient‐specific immune parameters.

**Methods:**

A comprehensive literature review was conducted, encompassing clinical trials, retrospective cohort studies, expert consensus guidelines, and population‐based data. Extracted outcomes included serological responses, infection‐related events, and vaccine safety in MM patients.

**Results:**

Patients with MM exhibit impaired vaccine responses due to hypogammaglobulinemia, T‐ and B‐cell dysfunction, and therapy‐induced lymphodepletion. Despite modest immunogenicity, influenza and pneumococcal vaccines reduce respiratory infections and hospitalizations. Sequential administration of PCV13 followed by PPSV23, as well as post‐autologous stem cell transplantation (ASCT) three‐dose regimens, is associated with reduced pneumonia incidence. COVID‐19 vaccines elicit variable responses, particularly in patients on anti‐CD38 or BCMA‐targeted therapies, highlighting the need for booster doses and, in selected cases, prophylactic monoclonal antibodies. Vaccines against herpes zoster, hepatitis B, and *Haemophilus influenzae* type B are also recommended, particularly around ASCT. Immunophenotypic markers such as CD19+ B‐cell and CD4+ T‐cell counts are predictive of vaccine responsiveness, supporting immune profiling as a tool for individualized vaccination planning.

**Conclusions:**

Vaccination remains a critical component of infection prevention in MM. Although immunogenicity may be attenuated, clinical benefits—namely, reduced infection burden and healthcare utilization—support broad vaccine implementation. A personalized approach, considering the treatment phase, disease control, and immune status, is essential to optimize vaccine effectiveness. Ongoing research into high‐dose, adjuvanted, and next‐generation vaccines is critical to enhance protection in this vulnerable population.

## 1 Introduction

Multiple myeloma (MM) is a hematological malignancy characterized by the clonal expansion of malignant plasma cells within the bone marrow, resulting in end‐organ damage such as osteolytic bone lesions, renal impairment, anemia, and—most critically—profound immunosuppression [1]. This immunodeficient state arises from both disease‐related mechanisms, including immunoparesis [2] and bone marrow infiltration, as well as treatment‐induced effects that compromise immune function [3].

A significantly increased risk of infection has been consistently reported in patients with MM. A population‐based study involving 9253 MM patients confirmed infections as a leading cause of morbidity and mortality in this population [4]. In one survey, 168 of 322 patients (approximately one in two) reported at least one infectious episode in the year preceding the initiation of therapy, while 135 of 314 patients (roughly two in five) experienced at least one infection within the first 6 months following treatment [5]. Respiratory infections—especially those caused by influenza and other respiratory viruses—frequently result in hospitalization, clinical deterioration, and treatment interruptions. Given the seasonal recurrence and high transmissibility of influenza, effective preventive strategies are of particular importance in immunocompromised individuals.

Vaccination remains a cornerstone of infection prevention in MM [6]. However, vaccine efficacy is often suboptimal due to the underlying immune dysfunction, which includes B‐ and T‐cell impairment, hypogammaglobulinemia, and a defective capacity to generate immunological memory. As a result, many MM patients fail to achieve adequate seroconversion or protective antibody titers following standard vaccination, necessitating consideration of alternative strategies—such as high‐dose formulation, booster doses, or adjuvanted vaccines—tailored to the patient's immune status.

The COVID‐19 pandemic further underscored the vulnerability of MM patients to viral infections. Studies show significantly increased risks of severe COVID‐19 outcomes, including hospitalization and death, irrespective of disease or treatment status [7]. Therapies targeting CD38 or B‐cell maturation antigen (BCMA) have been associated with attenuated humoral responses to SARS‐CoV‐2 vaccination, although some degree of cellular immunity may persist [8]. These findings highlight the need for comprehensive vaccination strategies, which may include booster doses, pharmacologic prophylaxis, and passive immunization in high‐risk subgroups [9].

Despite the strong clinical rationale, real‐world vaccine uptake among MM patients remains inconsistent [5]. Contributing factors include vaccine hesitancy driven by concerns about adverse effects and the proliferation of misinformation, often amplified through digital platforms. Clear, evidence‐based communication by healthcare providers is essential to counter misinformation, improve vaccine acceptance, and support informed decision‐making. Tailored educational initiatives in hematology care settings may further enhance patient engagement and facilitate shared decision‐making around infection prevention.

This review aims to synthesize current evidence regarding vaccination in MM, with a particular focus on the biological mechanisms underlying vaccine failure, clinical data on vaccine responses, and implications for clinical practice. By addressing key challenges and proposing evidence‐based solutions, we seek to support more effective and personalized immunization strategies for individuals living with MM.

## 2 Biological Background of Immunosuppression

Table 1 summarizes the multifaceted immune dysfunctions underlying the profound immunosuppression observed in patients with MM. These mechanisms—arising from both the disease itself and its treatments—contribute to a heightened risk of infection and diminished vaccine responsiveness.

**TABLE 1:** Biological background of immunosuppression in multiple myeloma.

Immunological component	Mechanism of dysfunction	Clinical implication
Humoral immunity (B cells)	Suppression of polyclonal immunoglobulin production (immunoparesis); depletion of normal plasma cells by malignant clones	Increased susceptibility to bacterial infections; impaired vaccine‐induced antibody production
Cellular immunity (CD4+ T cells)	Quantitative and functional depletion; impaired helper T‐cell response	Reduced coordination of immune responses; decreased vaccine efficacy
Regulatory T cells (Tregs) and MDSCs	Expansion of immunosuppressive subsets; inhibition of effector T and NK cells	Global suppression of immune surveillance and cytotoxic responses
Dendritic cells	Functional impairment in antigen presentation	Defective activation of naïve T‐cells; poor vaccine priming
Effect of treatments	Corticosteroids, proteasome inhibitors, IMiDs, anti‐CD38 antibodies deplete or impair lymphocyte function	Therapy‐induced immunodeficiency contributes to infection risk and diminished vaccine responses
Post‐ASCT immune reconstitution	Delayed recovery of B and CD4+ T‐cells (up to > 12 months posttransplant)	Limited immune memory and poor response to standard vaccinations

Immunosuppression in MM stems from both intrinsic disease‐related mechanisms and therapy‐induced immune alterations. The malignant proliferation of clonal plasma cells in the bone marrow disrupts normal hematopoiesis and suppresses polyclonal immunoglobulin production, leading to a condition known as immunoparesis. This is a defining feature of MM and involves broad immune dysfunction, including reduced production of uninvolved immunoglobulin, suppression of T and NK‐cell functions, and remodeling of the marrow microenvironment into an immunosuppressive niche [10].

Importantly, immunoparesis extends beyond simple hypogammaglobulinemia. T‐cell exhaustion is commonly observed, with upregulation of inhibitory receptors such as PD‐1/PD‐L1 on both myeloma cells and T cells, impairing cytokine production and cytotoxicity activity [11]. Unlike monoclonal gammopathy of undetermined significance (MGUS), immune dysfunction in MM is progressive, culminating in the loss of tumor‐specific cytotoxic response [12]. Regulatory T cells (Tregs) are also expanded and correlate with disease burden, further facilitating immune evasion [13].

The innate immune system is similarly compromised. NK cells become less effective due to altered cytokine balance and reduced activating ligands on MM cells [14]. Additionally, the accumulation of tumor‐associated macrophages and myeloid‐derived suppressor cells fosters an immunosuppressive marrow environment [15].

This immunological impairment not only predisposes patients to infections—particularly early in the disease or with high tumor burden—but also limits the efficacy of immunotherapies. Consequently, many current therapeutic strategies, including monoclonal antibodies, CAR‐T cells, and bispecific T‐cell engagers, are being designed to overcome or bypass this immune dysfunction [16, 17, 18, 19, 20].

Therapeutic regimens in MM significantly exacerbate immunosuppression. Agents such as proteasome inhibitors, corticosteroids, high‐dose melphalan, anti‐CD38 monoclonal antibodies (e.g., daratumumab, isatuximab), and cellular therapies like CAR‐T cells induce broad and sometimes prolonged depletion of B, T, and NK cells, impairing both humoral and cellular immunity [21, 22, 23, 24, 25]. Immunomodulatory drugs (IMiDs), such as lenalidomide, exert complex immunologic effects, enhancing certain immune functions while concurrently suppressing others.

High‐dose chemotherapy followed by autologous stem cell transplantation (ASCT) leads to profound and long‐lasting lymphodepletion. B‐cell recovery may take over a year, and reconstitution of CD4+ T cells is often even more delayed, severely compromising the ability to mount effective immune responses to pathogens or vaccines [6].

Additional factors—such as age‐related immune senescence, organ dysfunction, comorbidities, and prolonged exposure to immunosuppressive agents—further contribute to immune failure and infection risk [2]. T‐cell exhaustion associated with long‐standing therapy also plays a significant role in persistent immunodeficiency [24].

These overlapping mechanisms culminate in impaired serologic and cellular responses to vaccines. Standard immunization strategies often fail to confer adequate protection in MM patients. Therefore, a tailored vaccination approach is essential, incorporating disease status, treatment history, transplant timing, and immune biomarkers to improve vaccine efficacy and reduce infection‐related morbidity and mortality.

Overall, while vaccination remains strongly recommended for patients with MM, the limited efficacy of conventional immunization strategies underscores the urgent need for personalized vaccination protocols and further investigation into the immunological determinants of vaccine responsiveness in this uniquely immunocompromised population.

## 3 Infection and Vaccination in Multiple Myeloma

Tables 2 and 3 provide a structured and clinically practical overview of vaccination guidelines and determinants of vaccine responsiveness in patients with MM. Table 2 outlines the recommended vaccines against key pathogens, highlighting the importance of timing—particularly in relation to ASCT—and the need for tailored strategies based on immune recovery. Notably, it underscores the reduced serological response to influenza, pneumococcus, and SARS‐CoV‐2 in MM, with suggestions such as prime‐boost strategies or high‐dose formulations to improve efficacy.

**TABLE 2:** Recommended vaccinations in multiple myeloma patients.

Pathogen	Vaccine type	Timing and recommendations	Challenges/notes
Influenza A/B	Inactivated quadrivalent (standard or high‐dose)	Annually for all MM patients. Consider booster (prime‐boost) post‐ASCT or in poor responders.	Seroconversion suboptimal post‐ASCT; prime‐boost strategy may improve titers.
*Streptococcus pneumoniae*	PCV13 followed by PPSV23	Begin with PCV13, followed by PPSV23 ≥ 8 weeks later. Repeat PPSV23 after 5 years.	Response rates ~33%–40%; better if vaccinated pre‐ASCT or in remission.
SARS‐CoV‐2 (COVID‐19)	mRNA or adenovirus vector‐based	Recommended during disease control, ≥ 3 months post‐ASCT. Booster doses as needed.	Lower serologic response in patients on anti‐CD38 or BCMA therapies.
Herpes zoster (VZV)	Recombinant zoster vaccine (RZV, Shingrix)	Two doses, 2–6 months apart, especially if on proteasome inhibitors or post‐ASCT.	Live vaccines contraindicated.
Hepatitis B virus	Recombinant HBV vaccine (Engerix‐B, etc.)	For seronegative patients. Preferably before therapy or transplant.	Seroconversion rates < 50%; may need high‐dose or repeated regimens.
*Haemophilus influenzae*	Hib conjugate vaccine	One dose post‐ASCT or in asplenic patients.	Safe and well‐tolerated.
Tetanus, diphtheria, pertussis	Tdap followed by Td boosters	Complete series pre‐ASCT or during immune recovery.	Boosters every 10 years.
*Neisseria meningitidis*	MenACWY + MenB (conjugate vaccines)	Consider in patients post‐ASCT or with asplenia.	Not routinely used; assess individual risk.
HPV	Nonavalent HPV (Gardasil 9)	Optional in young adults with MM; follow general vaccination schedule.	Immunogenic and safe in immunocompromised adults.

**TABLE 3:** Determinants of vaccine response in multiple myeloma patients.

Factor	Effect on vaccine response
Disease status (CR/VGPR)	Higher response rates to influenza and pneumococcal vaccines.
Immunoparesis	Associated with impaired humoral responses and reduced seroconversion.
CD19+ B‐cell count	Strong predictor of effective serologic response.
CD4+ T‐cell count	Associated with T‐dependent antibody responses; predictive of influenza vaccine efficacy.
Recent high‐dose chemotherapy/ASCT	Linked to reduced vaccine efficacy; booster vaccination may be needed.
Active treatment with anti‐CD38	Attenuates humoral responses to SARS‐CoV‐2 and other vaccines.
Timing of vaccination	Best responses during remission or ≥ 3–6 months post‐ASCT.
Vaccine formulation (e.g., adjuvanted, high‐dose)	May enhance immunogenicity, but more evidence is needed.

Table 3 complements this by identifying critical immunological and treatment‐related factors that influence vaccine outcomes. Factors such as disease remission status, immune cell counts (CD4+ and CD19+), and recent exposure to intensive therapies like ASCT or anti‐CD38 antibodies are key predictors of vaccine success. Together, these tables emphasize the need for individualized vaccination protocols in MM, guided by immunological biomarkers and clinical context, rather than a one‐size‐fits‐all approach. They offer a strong foundation for implementing evidence‐based vaccination strategies to mitigate infectious risks in this highly vulnerable population.

### 3.1 Pneumococcal Infection and Vaccination

The immunocompromised state of patients with MM predisposes them to a wide range of infections, with invasive pneumococcal disease (IPD) being particularly concerning due to its high morbidity and mortality. *Streptococcus pneumoniae* , the causative pathogen of IPD, can lead to severe outcomes such as pneumonia, meningitis, and sepsis, especially in immunocompromised individuals.

Epidemiological data consistently show that patients with hematological malignancies, including MM, are at significantly higher risk for IPD compared to the general population. One retrospective study from Alberta, Canada, reported an annual incidence of 673.9 IPD cases per 100 000 individuals with MM, compared to 11.0 per 100 000 in the general adult population—an odds ratio of 62.8 (95% CI: 39.6–99.8; *p* < 0.001) [26]. A large population‐based cohort study from the Netherlands confirmed these findings, showing markedly elevated IPD incidence among hematological malignancy patients, with MM representing one of the highest‐risk groups [27]. The study also noted that childhood pneumococcal vaccination programs contributed to decreased IPD incidence in immunocompromised adults through herd immunity, although the burden in MM remains substantial.

The clinical implications are clear: pneumococcal vaccination must be a core element of infection prevention in MM. *S. pneumoniae* is the leading cause of community‐acquired pneumonia and often results in prolonged hospitalization and high mortality among MM patients [28].

The 13‐valent pneumococcal conjugate vaccine (PCV13) and the 23‐valent pneumococcal polysaccharide vaccine (PPSV23) are routinely recommended in MM. PPSV23 elicits protective antibody responses in approximately 33%–40% of patients, with better responses observed when administered before ASCT or during remission. The three‐dose PCV13 regimen, especially in the posttransplant setting, has demonstrated serological response rates of up to 58%, and concurrent lenalidomide maintenance therapy does not appear to adversely affect vaccine efficacy [29].

A recent expert consensus using a modified Delphi method strongly endorsed sequential vaccination with PCV13 followed by PPSV23. This approach maximizes serotype coverage and enhances immunogenicity. The panel emphasized tailoring vaccination to the patient's treatment phase and immune status, while promoting greater awareness among healthcare providers to overcome barriers to vaccine uptake [29].

Recently, a retrospective study involving 38 MM patients undergoing ASCT between 2020 and 2023 was conducted to compare the traditional PCV13 + PPSV23 vaccination schedule with a three‐dose regimen of PCV20. An overall serotype response rate of 44.4% was reported. Notably, PCV20 was associated with improved immunogenicity against serotypes 8, 10A, 11A, 12f, 15B, 22f, and 33f, regardless of baseline immunoglobulin levels, absolute CD4+ T cell count, or type of maintenance treatment [30].

Furthermore, in June 2024, the US FDA approved PCV21, a novel pneumococcal conjugate vaccine that includes 21 serotypes—eight of which are not covered by any currently available pneumococcal vaccines. In a large international phase 3 trial (STRIDE‐3), PCV21 demonstrated favorable safety and tolerability profiles, with robust immunogenicity against the 10 serotypes shared with PCV20 and superior responses to the 11 unique serotypes [31].

Despite these benefits, challenges remain. Vaccine responsiveness is frequently attenuated due to immunoparesis and B‐cell depletion from therapies such as anti‐CD38 monoclonal antibodies and proteasome inhibitors. The resulting immune deficits may necessitate individualized vaccine schedules and repeated boosters. Additionally, the timing of vaccination relative to immunosuppressive therapy or HSCT remains under investigation.

Nevertheless, the risk–benefit ratio favors vaccination. When integrated into a personalized immunization strategy, pneumococcal vaccines significantly reduce the incidence of pneumonia and improve clinical outcomes in MM patients.

### 3.2 Comprehensive Viral Vaccination Strategies in MM: Evidence, Efficacy, and Recommendations

Influenza viruses, members of the Orthomyxoviridae family, are major contributors to seasonal epidemics and sporadic pandemics worldwide. Influenza A virus (IAV) is particularly pathogenic, accounting for 3 to 5 million cases of severe illness and up to 650 000 respiratory deaths annually, according to the World Health Organization. Its segmented, negative‐sense RNA genome, along with high mutation rates in hemagglutinin and neuraminidase surface proteins, allows for frequent antigenic drift and shift, promoting immune escape and facilitating the emergence of pandemic strains. Clinical manifestations of influenza range from mild respiratory symptoms to life‐threatening pneumonia, acute respiratory distress syndrome, and secondary bacterial infections. Vulnerable populations—including the elderly, infants, pregnant women, and immunocompromised individuals—are at the highest risk for severe outcomes. Annual influenza vaccination, guided by global surveillance and updated formulations, remains the most effective preventive strategy [32].

The Cochrane systematic review by Cheuk et al. evaluated the efficacy of viral vaccines in immunocompromised patients with hematological malignancies, including five randomized controlled trials (RCTs) on inactivated influenza vaccines [33]. Despite attenuated serologic responses, influenza vaccination reduced infection rates and hospitalizations, justifying routine use in this high‐risk group.

Among patients with MM, significant deficiencies in T‐cell, B‐cell, and antigen‐presenting cell (APC) functions lead to high susceptibility to infections. In an RCT included in the Cochrane review, influenza vaccination in MM patients undergoing chemotherapy led to a 61% reduction in lower respiratory infections and an 83% reduction in hospitalizations. These findings are clinically significant, given the risks of treatment delays and mortality due to respiratory infections in MM.

Although serological responses were diminished, especially in those undergoing therapy, clinical protection was still observed. Adverse events were generally mild and self‐limited. While two‐dose strategies have been evaluated, data remain inconclusive regarding their superiority over single‐dose regimens.

A recent retrospective single‐center study further evaluated immune responses to standard versus prime‐boost influenza vaccination in 71 MM patients during the 2019–2020 season [34]. While a booster increased antibody titer, it did not significantly enhance response rates. Multivariate analysis revealed that patients in complete or very good partial response (CR/VGPR) and those without immune paresis were more likely to achieve adequate serological protection. Higher CD19+ B‐cell and CD4+ T‐cell counts also correlated with vaccine responsiveness. Importantly, patients closer to HSCT showed reduced responses, emphasizing the need to time vaccination post‐immune reconstitution. These data underscore the importance of individualized vaccination strategies in MM. Patients in remission may benefit from standard vaccination, whereas those early post‐HSCT may require booster strategies. Immune profiling may guide decisions on vaccine timing and regimen intensity.

Additional strategies to improve vaccine efficacy in immunocompromised patients, including those with MM, involve the use of high‐dose and adjuvanted influenza vaccines. High‐dose influenza vaccines contain four times the amount of hemagglutinin antigen per strain compared to standard‐dose formulations. This increased antigen content has been shown to elicit stronger antibody responses and may enhance protection in immunocompromised individuals. Notably, in a large US veteran population, high‐dose vaccines were associated with reduced hospitalization costs for respiratory and cardiovascular complications over five influenza seasons, underscoring their clinical and economic value [35]. Similarly, adjuvanted influenza vaccines (aIIV), which incorporate the MF59 adjuvant, have demonstrated enhanced immunogenicity. MF59 promotes a more robust immune response by recruiting and activating APCs and augmenting T‐cell‐mediated immunity. Recent evidence in patients with hematologic malignancies indicates that aIIVs are capable of generating stronger and more durable immune responses compared to standard formulations [36].

In addition to influenza and pneumococcal immunization, patients with MM should receive a broad spectrum of other vaccines to mitigate their heightened risk of infection [33]. Patients with MM are at increased risk of hepatitis B virus (HBV) reactivation, particularly in the context of immunosuppressive therapy or HSCT. Reactivation can occur even in patients with resolved HBV infection (anti‐HBc positive, HBsAg negative), leading to hepatitis flares and potentially liver failure. Vaccination against HBV is recommended for all seronegative MM patients, ideally before initiation of therapy or HSCT. However, vaccine response rates in MM are suboptimal, with seroconversion rates ranging from 20% to 50%, depending on disease status and concurrent treatments. High‐dose or double‐dose HBV vaccines and booster regimens may improve seroprotection in this population, although more studies are needed to define optimal strategies. Routine screening for HBV serological markers and antiviral prophylaxis in at‐risk patients is also essential.

The incidence of herpes zoster is significantly elevated in MM patients, particularly among those receiving proteasome inhibitors or undergoing HSCT. The use of bortezomib and other novel agents such as daratumumab or carfilzomib has been associated with a threefold to fivefold increase in zoster reactivation. The recombinant zoster vaccine (RZV, Shingrix)—a nonlive, adjuvanted vaccine—has demonstrated efficacy and safety in immunocompromised populations and is the preferred option over the live‐attenuated vaccine, which is contraindicated in this setting. Vaccination is recommended for all patients 18 years or older with a higher risk of HZ R1A2. Two doses, spaced 2–6 months apart, are required. A recent systematic review and meta‐analysis evaluating the immunogenicity of the RZV provided compelling evidence of its efficacy, even in immunocompromised populations. By analyzing both humoral and cell‐mediated immune responses post‐vaccination, the study demonstrated that RZV induces robust and durable immunogenicity. Notably, the analysis confirmed that RZV maintains its effectiveness across various patient populations with immunocompromising conditions, suggesting its potential to overcome immune suppression commonly seen in hematologic malignancies such as MM [37]. Preliminary data suggest good immunogenicity even in patients receiving lenalidomide or maintenance therapy, though real‐world effectiveness studies in MM are ongoing.

Immunity to tetanus and diphtheria can wane over time, and pertussis outbreaks remain a public health concern. MM patients, particularly those undergoing HSCT, should receive DTPA vaccination if not previously immunized, followed by decennial Td boosters. Immunization should ideally be completed before transplant or during recovery to ensure adequate T‐cell priming. Although data specific to MM are limited, these vaccines are inactivated and considered safe and well‐tolerated in immunocompromised individuals [33].

*Haemophilus influenzae* type B remains a potential cause of severe invasive disease in immunocompromised hosts. Hib vaccination is particularly recommended post‐HSCT, as part of the re‐immunization protocol. A single dose may be sufficient for adults without prior immunization; however, in cases of profound immunodeficiency, repeat dosing may be necessary. Conjugate formulations (e.g., ActHIB) are preferred due to better T‐cell‐dependent immune responses.

Although not routinely to be carried out, meningococcal vaccination is recommended by the Advisory Committee on Immunization Practices (ACIP) in MM patients, especially in those undergoing HSCT or with additional risk factors such as functional or anatomical asplenia. Quadrivalent conjugate vaccines (MenACWY) and serogroup B vaccines (MenB) are both inactivated and safe for use. Vaccination should ideally be completed prior to transplant, with re‐vaccination as per guidelines depending on age and risk [33].

While not currently standard for all MM patients, HPV vaccination may be considered in younger adults with MM to reduce the long‐term risk of HPV‐associated malignancies. The nonavalent HPV vaccine (Gardasil 9) is inactivated and well‐tolerated in immunocompromised individuals. Its use should follow general adult vaccination guidelines, with consideration of individual risk profiles and ongoing therapy [33].

Patients with MM benefit from a comprehensive immunization strategy that extends beyond influenza and pneumococcal vaccines. Vaccines against hepatitis B, herpes zoster, tetanus–diphtheria–pertussis, Hib, meningococcus, and potentially HPV are integral components of infection prevention, particularly in the context of HSCT or novel therapies. Given the suboptimal vaccine responses in MM, strategies such as early vaccination, higher antigen doses, or booster regimens may be warranted. Clinical decisions should be individualized based on disease status, treatment timing, and immune reconstitution. Continued research and real‐world studies will be essential to refining these recommendations and improving long‐term outcomes for patients with MM. In conclusion, vaccination remains central to infection prevention in MM. Despite immunological barriers, clinical benefits—especially reductions in infection, hospitalization, and mortality—justify their routine use. Personalized approaches based on disease status, therapy phase, and immune profiling are essential to optimizing outcomes.

## 4 Discussion

Patients with MM face an exceptionally high burden of infectious morbidity due to a confluence of intrinsic immune dysfunction and treatment‐related immunosuppression. This vulnerability extends across a broad spectrum of pathogens, including *S. pneumoniae* , influenza viruses, varicella‐zoster virus (VZV), HBV, and most recently, SARS‐CoV‐2 [8, 38, 39]. The clinical consequences of these infections are substantial, often resulting in hospitalizations, treatment delays, and increased mortality. Consequently, vaccination has emerged as a cornerstone of infection prevention in MM and is now considered an integral component of supportive care.

The available evidence underscores that, despite attenuated serological responses—particularly in patients receiving proteasome inhibitors, IMiDs, anti‐CD38 monoclonal antibodies, or undergoing ASCT [9]—clinically meaningful protection can still be achieved. This has been most clearly demonstrated with influenza and pneumococcal vaccines, where reductions in lower respiratory tract infections, hospitalizations, and pneumonia incidence have been consistently reported. The immunogenicity of pneumococcal conjugate (PCV13) and polysaccharide (PPSV23) vaccines, while suboptimal in some cases, is sufficient to justify their inclusion in routine clinical protocols, especially when administered in a sequential and timely manner [28, 29].

Importantly, emerging evidence indicates that vaccine responsiveness in MM is not uniform but is highly heterogeneous and closely associated with the extent of immune reconstitution. In patients undergoing allogeneic hematopoietic stem cell transplantation (allo‐HSCT), the vaccination strategy plays a pivotal role in re‐establishing immunological memory and protecting against serious infections. This is particularly crucial given that allo‐HSCT leads to the loss of pre‐existing immunity—whether naturally acquired or vaccine‐induced—and induces a prolonged period of immunodeficiency. In this vulnerable subset, the administration of live attenuated vaccines should be strictly avoided for at least the first 2 years posttransplant, and even longer in the presence of GVHD [40].

Current recommendations from the European Society for Blood and Marrow Transplantation (EBMT) emphasize the need to reinitiate the full vaccination schedule starting 3 to 6 months posttransplant. The guidelines advocate for the use of highly immunogenic pediatric vaccine formulations to enhance immunoprotection during immune reconstitution, suggesting the repetition of the entire vaccine schedule, using more immunogenic pediatric formulations and starting 3–6 months posttransplant [40]. On the other hand, patients in complete remission or very good partial response, and those with higher CD19+ B‐cell and CD4+ T‐cell counts, are more likely to mount adequate vaccine‐induced immunity. This finding highlights the critical importance of individualized vaccination strategies tailored to the patient's immunologic profile and treatment timeline.

The COVID‐19 pandemic has further exposed the immunologic fragility of MM patients. Diminished seroconversion rates and rapid waning of protective antibodies after SARS‐CoV‐2 vaccination are well documented, especially in patients undergoing active therapy or post‐ASCT [8, 9]. Booster doses and passive immunization strategies (e.g., monoclonal antibodies) are increasingly recognized as necessary adjuncts for those unable to generate robust vaccine responses. Emerging variants and the decline in neutralizing antibodies over time also justify the consideration of regular (potentially annual) revaccination strategies in high‐risk populations.

While safety profiles of vaccines remain favorable in MM, significant knowledge gaps persist. The optimal dosing regimens and predictive value of immune biomarkers require further investigation. Moreover, real‐world effectiveness studies remain limited and should be prioritized to validate and refine current recommendations.

Vaccination in MM is not simply a preventive adjunct—it is a critical element of disease management. As therapies for MM become increasingly effective and complex, patients are living longer but often under sustained immunosuppression. This prolonged immunocompromised state necessitates a proactive and nuanced approach to infection prevention.

In clinical practice, vaccine decisions should be individualized and guided by three key considerations: timing, immune status, and treatment phase. Where feasible, vaccinations should be administered during periods of disease stability, ideally before initiating immunosuppressive therapy or at least 3–6 months post‐ASCT to allow for immune reconstitution. In patients receiving BCMA‐targeted agents or anti‐CD38 therapies, clinicians should anticipate suboptimal vaccine responses and consider supplemental strategies such as booster dosing or passive immunization.

CAR‐T therapy and bispecific antibodies have become essential components of the therapeutic landscape for relapsed/refractory MM. Vaccinations in patients receiving these therapies pose a critical challenge due to the profound and prolonged immunosuppression induced by treatment. This immune compromise is characterized by T‐cell dysfunction, B‐cell aplasia, and sustained hypogammaglobulinemia, all of which increase susceptibility to infectious complications. Vaccinations against influenza, pneumococcal, hepatitis B, HZ, tetanus‐diphtheria‐pertussis (Tdap), *Haemophilus influenzae* type b (Hib), meningococcus, and, when indicated, human papillomavirus (HPV) are strongly recommended (Table 4). Ideally, these vaccines should be administered 2–4 weeks before the initiation of CAR‐T or bispecific antibody therapy to maximize immunogenicity. Post‐treatment, re‐vaccination should be considered beginning 3–6 months after therapy, based on recovery of B‐ and T‐cell populations and normalization of immunoglobulin levels. Live attenuated vaccines remain contraindicated in this context and should be deferred for at least 24 months following therapy, or until there is evidence of full immune reconstitution. While intravenous immunoglobulin (IVIG) replacement therapy is often warranted to provide passive protection during periods of severe hypogammaglobulinemia, it is not a substitute for active immunization and should be viewed as a complementary strategy [40].

**TABLE 4:** Vaccinations and relative timing recommended in multiple myeloma patients treated with CAR‐T and bispecific antibodies.

Vaccine	Start timing after CAR‐T and bispecific antibodies (months)	Notes
Influenza	3–6	Repeat annually Recommended high dose or adjuvanted formulations
Covid‐19	≥ 3	Regular boosters recommended
Pneumococcal	≥ 3	
DTPA	≥ 6	Pediatric formulation (improvement of response)
Hib, HBV, meningococcus	≥ 3–6	Check titers
Live vaccines (measles, mumps, rubella, and VZV)	≥ 24	To be administered only when immune reconstitution is achieved

Routine annual influenza vaccination should be strongly encouraged for all MM patients, regardless of treatment status. Prime‐boost strategies may benefit selected subgroups, particularly those recently transplanted or with impaired immune profiles. Pneumococcal and recombinant zoster vaccines should be administered sequentially and in alignment with transplant schedules. For HBV, Hib, Tdap, and meningococcal vaccinations, a comprehensive and updated immunization history is essential, and gaps should be addressed proactively.

Beyond vaccine administration, clinicians must also address vaccine hesitancy—a growing concern fueled by misinformation and lack of disease‐specific education. Tailored communication and shared decision‐making are essential to improving adherence and building patient confidence. Laboratory evaluation of immune parameters, such as CD4+ and CD19+ counts, may provide a valuable tool for anticipating response and guiding vaccine timing.

Looking forward, integration of immune profiling, expanded use of adjuvanted formulations, and incorporation of novel platforms such as mRNA‐based vaccines may transform the vaccination landscape for MM. Continued collaboration between hematologists, immunologists, and infectious disease specialists will be key to optimizing outcomes. As the infection‐related risks in MM are unlikely to abate, particularly in the era of advanced therapies, the role of vaccination will only grow in importance.

Despite growing interest in vaccination strategies for MM, several limitations persist in the current body of evidence. Most studies evaluating vaccine efficacy in MM are observational or retrospective, with a relative paucity of RCTs, particularly for high‐dose or adjuvanted vaccine formulations. As a result, the optimal antigen dose, booster schedules, and comparative effectiveness of different vaccine platforms remain incompletely defined.

Moreover, clinical trials often underrepresent patients with relapsed/refractory MM or those undergoing advanced therapies such as CAR‐T cells or bispecific antibodies. These subgroups, among the most immunosuppressed, may respond differently to vaccination, yet data on immunogenicity and clinical protection in this population are limited.

Another key limitation lies in the heterogeneity of immunogenicity assessment across studies. Different methodologies—including variable timing of antibody measurements, use of distinct assays (e.g., ELISA, neutralization tests), and lack of standardized sero‐protection thresholds—hamper direct comparisons and meta‐analytic synthesis.

Finally, most available studies emphasize humoral responses, while data on cellular immunity—particularly T‐cell responses—remain scarce, despite their growing relevance in immunocompromised hosts. These limitations underscore the urgent need for prospective, well‐designed studies incorporating both serologic and cellular endpoints, particularly in populations receiving modern MM therapies.

In sum, a well‐structured, patient‐specific vaccination plan is not optional but essential. Proactive immunization, informed by evidence and individualized risk, represents a powerful tool in reducing preventable complications and improving the quality and longevity of life in patients with MM.

## Author Contributions

All authors contributed to the manuscript and were involved in revisions and proofreading. All authors approved the submitted version.

## Conflicts of Interest

The authors declare no conflicts of interest.

## Data Availability

Data sharing is not applicable to this article as no new data were created or analyzed in this study.
